# Omalizumab as successful treatment option in severe peanut allergy

**DOI:** 10.1186/2045-7022-3-S3-P20

**Published:** 2013-07-25

**Authors:** E Dehlink, C Bannert, T Eiwegger, S Diesner, S Gruber, Z Szépfalusi

**Affiliations:** 1 Department of Pediatrics and Adolescent Medicine, Medical University Vienna, Vienna, Austria

## Background

Peanut allergy is a potentially life threatening condition, which usually persists lifelong. Allergen avoidance is challenging as reactions often occur to traces of peanut in processed foods.

## Methods

We present the case of a girl, who was diagnosed with peanut allergy at age 8 after she developed abdominal pain, urticaria and angioedema following a meal of cornflakes with peanut chips. No food challenge was performed at that time, and she was advised to avoid peanuts. Despite avoiding foods visibly containing peanut, she repeatedly experienced urticaria, burning of the tongue, nausea, and vomiting after consumption of different chocolates and ice creams. At age 13, she experienced a first episode of dyspnea and wheezing after eating a cake. This was when she was presented to our outpatient clinic.

## Results

To confirm peanut allergy and to determine the allergen threshold dose, we performed open peanut challenge, during which she developed anaphylaxis that required epinephrine at a dose of 1.5g of peanut, corresponding to one peanut. Because of the severity of the reaction and the repeated incidents despite elimination diet, we decided for a treatment with omalizumab, at a dose of 300mg every 4 weeks for 1 year, which was tolerated well by the patient. Four weeks after the last dose we repeated the open peanut challenge. The girl tolerated all doses with a cumulative dose of 5g, corresponding to 3 peanuts, without any symptoms. Total- and peanut-specific IgE levels remained unchanged, whereas titrated skin prick test reactivity slightly decreased. As a bystander effect, the girl’s cat- and grass pollen allergy resolved completely.

## Conclusion

Omalizumab is an effective and safe treatment option in severe peanut allergy.

## Disclosure of interest

None declared.

